# Mannan oligosaccharides as a prebiotic for laying hens: effects on fertility, hatchability, productive performance, and immunity

**DOI:** 10.1093/tas/txae123

**Published:** 2024-08-23

**Authors:** Islam M Youssef, Ahmed K Aldhalmi, Shatha G Felemban, Ahmed I Elsherbeni, Hassan A Khalil, Magdy S Hassan, Haiam S Abd El Halim, Mohamed E Abd El-Hack, Khaled M Youssef, Ayman A Swelum, Vincenzo Tufarelli, Maher A Abo-Samra

**Affiliations:** Animal Production Research Institute, Agriculture Research Center, Dokki, Giza 12618, Egypt; College of Pharmacy, Al- Mustaqbal University, 51001 Babylon, Iraq; Medical Laboratory Sciences Department, Fakeeh College for Medical Sciences, Jeddah 21461, Saudi Arabia; Animal Production Research Institute, Agriculture Research Center, Dokki, Giza 12618, Egypt; Animal Production Department, Faculty of Agriculture, Suez Canal University, Ismailia 41522, Egypt; Animal Production Research Institute, Agriculture Research Center, Dokki, Giza 12618, Egypt; Animal Production Department, Faculty of Agriculture, Suez Canal University, Ismailia 41522, Egypt; Poultry Department, Faculty of Agriculture, Zagazig University, Zagazig, 44511, Egypt; Food Technology Department, Faculty of Agriculture, Suez Canal University, Ismailia 41522, Egypt; Department of Animal Production, College of Food and Agriculture Sciences, King Saud University, Riyadh 11451, Saudi Arabia; Department of Precision and Regenerative Medicine and Jonian Area, Section of Veterinary Science and Animal Production, University of Bari Aldo Moro, 70010 Valenzano, Bari, Italy; Animal Production Department, Faculty of Agriculture, Suez Canal University, Ismailia 41522, Egypt

**Keywords:** carcass, egg production, estradiol-17β, immune response, Mannan oligosaccharides

## Abstract

This experiment examined how adding mannan-oligosaccharides (MOS) to the diet affected fertility, hatching rates, egg production, carcass characteristics, cost-effectiveness, and immune function in laying hens. One hundred and twenty Mandarah chickens (30 hens and 3 roosters per group) were randomly chosen between 34 and 50 wk old and divided into four groups. The first group was the control group, which was given just the basal diet. The basal diet was given to the second, third, and fourth experimental groups along with three different levels of MOS (0.1, 0.2, and 0.5 g/kg diet, respectively). Results found that hens fed MOS at various levels laid eggs at a significantly higher rate, enhanced egg number, egg mass and feed conversion ratio than the control group (*P* < 0.05). MOS seemed to improve carcass quality. The best results for egg quality (Haugh unit) and testosterone levels were seen with a dose of 0.5 g/kg of MOS compared to the control birds (*P* < 0.05). All MOS levels led to higher estradiol-17β (E2) levels and better economic efficiency (EE). MOS also improved the hens’ immune systems as compared to the control group. Hens-fed MOS had significantly greater levels of antibodies against Influenza viruses (H9N2) and Infectious Bronchitis Virus (*P* < 0.05). Also, the spleen and thymus gland, both crucial immune system components, were slightly larger (*P* < 0.05). It’s important to note that fertility rates, hatchability, and embryo mortality rates remained similar across all groups. So, our findings suggest that incorporating MOS into the birds’ diet enhances their productivity, strengthens their immune system, improves EE, and contributes to the overall health of the hens.

## Teaser

This work examined how adding mannan-oligosaccharides (MOS) to the diet affected fertility, hatching rates, egg production, carcass characteristics, cost-effectiveness, and immune function in laying hens. Findings suggested that MOS enhances productivity, strengthens the immune system, improves economic efficiency, and contributes to the overall health of the hens.

## Graphical Abstract

**Graphical Abstract:**
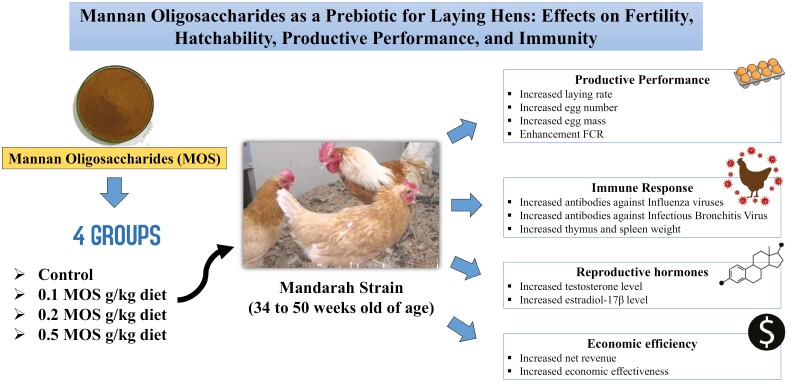

## Introduction

Egg production is expected to continue expanding because global demand for chicken eggs is expected to increase by 39% between 2005 and 2030 (MacLeod et al., 2013; Salami et al., 2022; Rafed et al., 2024). The egg industry faces challenges in raising chickens sustainably, ethically, economically, and affordability, but innovative solutions can be developed by considering chicken diet and raising methods (De Olde et al., 2020). In the same vein, the use of alternatives to antibiotics in feed may reduce antibiotic encounters while maintaining the health and well-being of animals and enhancing their productivity (Yang et al., 2009; Callaway et al., 2021; Youssef et al., 2023a, 2024). Researchers are developing new feed additives to enhance farm animals’ reproductive performance, productivity, and overall health, with natural additives like probiotics, prebiotics, organic acids, phytochemicals, and enzymes being the safest options (Abd El-Hack et al., 2021; Youssef et al. 2023b).

Mannan oligosaccharide is a special sugar found in the cell walls of yeast called *Saccharomyces cerevisiae* (Lepczyński et al., 2024). To release MOS, yeast cells are broken open using various techniques, including chemicals, physical procedures, and even enzymes. The purification involves treating the mixture with an alkaline solution, spinning it in a centrifuge, and applying a drying spray. Procedures like spray drying and membrane filtration can further purify and concentrate MOS. The finished result is a light brown MOS powder (González et al., 2023).

Research shows that supplementing chicken feed with MOS offers a natural alternative to antibiotics. MOS appears to boost the immune system and improve nutrient absorption in chickens (Spring et al., 2015; Youssef et al., 2023c). According to Corrigan et al. (2017), MOS’s ability to attach and stop pathogens from colonizing the gut is partially responsible for these enhancements. Numerous thorough evaluations conducted on laying hens and broiler breeders revealed that MOS can enhance impregnability, performance, and eggshell condition in several ways (Shashidhara and Devegowda, 2003; Gurbuz et al., 2011; Adli et al., 2023). MOS may enhance intestine structure, gut bacteria balance, and digestive enzyme function, potentially enhancing nutrient absorption and digestion (Yang et al., 2008; Li et al., 2023). MOS, a natural alternative to antibiotic growth promoters, promotes healthy growth and acts as a prebiotic, supporting gut health and reducing harmful bacteria growth (Yaqoob et al., 2021; Rajesh et al., 2024), boosting immune performance in broilers (Wang et al. 2003; Kyoung et al., 2023), then improve productive performance of laying hens (Sheoran et al., 2018; Youssef et al., 2023a).

Researchers have extensively studied how MOS acts as a powerful binding site for bacteria. MOS competes with the natural docking stations on the intestinal cells. This clever trick fools pathogens with special attachments (mannose-specific type-1 fimbriae) into latching onto the MOS instead. As a result, these harmful bacteria pass through the gut without causing any trouble (Conlon et al., 2022; Tsiouris et al., 2023).

In addition, MOS has potential applications, such as being a feed additive to promote the reproductive functioning of poultry (Iqbal et al., 2017). MOS could improve production performance and egg quality when utilized as a replacement for antibiotics in support of laying hens (Hassan et al., 2015). Similarly, Jahanian and Ashnagar (2015) found that adding MOS to bacterial-infected layers increased EM and production, making MOS a beneficial feed additive and a substitute for antibiotics.

So, exploring the potential effects of MOS on the productive performance and immune response of locally bred laying hen strains is essential to see if these nutritional additives can improve the productivity and health of these local laying hen breeds. Therefore, this experiment aimed to examine the influences of mannan oligosaccharides dietary supplementation on the mortality rate, economic efficiency (EE), and reproductive performance of mandarah strain hens.

## Materials and Methods

### Ethical Statement

The regulation 2010/63/EU of the European Parliament and of the Council of September 22, 2010, and EC No 767/2009, which was adopted on July 13, 2009, on safeguarding animals used for research purposes is cited by the authors as the source for the processes that were placed on the animals. The care of the birds used in this experiment and the experimental methods followed the Animal Care and Ethics Committee of the Animal Production Department, Faculty of Agriculture, Suez Canal University, Ismailia, Egypt.

### Birds and Management

A total of 120 hens and 12 roosters from the Mandarah strain, all 34 wk old, were divided randomly into four groups. The Mandarah chicken, well-suited to Egypt’s climate, is a unique breed developed over 8 yr. Breeders crossed Alexandrian line males with Dokki four females for four generations, specifically selecting for egg production.

All the hen groups showed similar daily egg production and average weight (around 46.45% and 1,428.56 g, respectively). The hens were kept under consistent hygiene and management practices. Their basal laying diet followed the National Research Council guidelines from 1994. The specific ingredients and nutrient breakdown of this diet are shown in Table 1. The hens received 16 h of light and 8 h of darkness throughout the experiment daily. The average temperature during the experiment was a comfortable 23.51 °C, with a relative humidity of 58.81%. The chickens were housed in semi-closed housing systems with floor-laying pens measuring 2 m by 1.5 m. The experiment monitored egg production for 16 wk, from when the hens were 34 wk old until they reached 50 wk.

**Table 1.** Components and the chemical composition of the basal diet

Ingredients	%
Yellow corn (8.5 %)	63.14
Soybean meal (44 %)	27.10
Limestone	7.60
Dicalcium phosphate	1.50
Dl-Methionine 99%	0.06
NaCl	0.3
Vit. + Min. premix^*^	0.3
Calculated analysis^†^	
Crude protein, %	17.33
ME, kcal/kg	2722
Calcium, %	3.35
Available phosphorus, %	0.40
Lysine, %.	0.88
Methionine, %	0.34
Methionine + Cystine%	0.64
Zinc (mg/kg)	22.21

^*^Vitamins and minerals premix: Given per Kg of diet: Vitamin A, 12000 IU; Vitamin E, 10 mg; Vitamin D3, 2200 ICU; Vitamin K3, 2 mg; Vitamin B1, 1 mg; Vitamin B2 5 mg; B6 1.5 mg; B12 10 mcg; Pantothenic acid 10 mg; Nicotinic acid 30 mg; Folic acid 1 mg, Biotein 1.5 mcg; Choline 250 mg; Copper 10 mg; Selenium 0.1 mg; Cobalt 0.1 mg Manganse 60 mg; Iron 30 mg; Zinc 50 mg; Iodine 1 mg.

^†^Calculated according to NRC (1994).

### Experimental Design

Randomly divided individually weighed birds (30 hens and 3 roosters each) into four equal groups. Each group received a single treatment, randomly assigned to three identical replicates. These pens, each containing 10 hens and one rooster, served as the experimental unit. The first group, acting as the control, received a basal diet. The remaining groups received the same basal diet supplemented with increasing levels of MOS: 0.1, 0.2, and 0.5 g/kg for the second, third, and fourth groups, respectively. ALLTECH, USA, was the supplier of mannan-oligosaccharides.

## Measurements

### Live Body Weight

At the beginning of the experiment, when birds were at 34 wk old, and again at the end at 50 wk old, all the birds were weighed individually. The changes in the birds’ body weight across all experimental groups were calculated.

### Egg Production Traits

The laying rate (LR) in every replicate inside each experimental group was estimated throughout the experiment and the egg number (EN) was recorded every day. Eggs were individually weighted (EW) two times a week to the closest gram. Furthermore, every experimental group’s egg mass (EM) was determined. Moreover, weekly records of each replicate’s feed consumption (FC) and feed conversion ratio (FCR) were made (Youssef et al., 2023a).

### Egg Quality Traits

A collection of 432 eggs at the ages of 38, 42, 46, and 50 wk (4-wk intervals) were utilized to investigate different aspects of egg quality. From every treatment, fresh eggs were gathered, weighed, and divided. The albumen height, yolk height, egg width and length, and diameter were all measured with a digital calibrator. Utilizing an accurate scale, the weighing of the yolks, the height and weighing of the albumen, and the weighing of the shell were all measured. A micrometer (Series 102, MISUMI (THAILAND) CO., LTD., manufactured by MITUTOYO) was used to determine the shell thickness involving the membranes. The egg shape index (ESI) was determined by taking the highest egg width and the greatest egg length and multiplying the result by 100. (Anderson et al., 2004). The height of egg yolk and albumen was determined with a digital height gauge (Single Column Digital Height Gauge, Yamer), and the yolk diameter and albumin length and width were determined with a digital caliper (Mutitoyo, Japan). Height/diameter × 100 was utilized to estimate the yolk index (Kondaiah et al., 1983). In accordance with Moula et al. (2013), the Haugh unit (HU) score for every egg was determined as follows: (H + 7.57 − 1.7 W^0.37^) = 100 log where W = egg weight (gm); H = albumen height (m.m.).

### Carcass Characteristics

At the end of the experiment, nine birds were selected randomly from each treatment group at 50 wk. The selected birds were given a 12-h fast before being slaughtered. They were then separately weighed alive to the closest gram and slaughtered with a sharp knife in accordance with Islamic procedure, which involves severing the throat close to the first vertebrae and the jugular vein. Following full bleeding, the birds’ heads, shanks, and plucks were removed to weigh and dress them. The gut, gizzard, lungs, spleen, thymus gland, fat from the abdomen, liver, heart, and reproductive organs were removed after the birds were eviscerated. Weighing was done independently for the carcass, belly fat, and giblets (empty gizzard, liver, and heart) (Baéza et al., 2022).

### Fertility, Hatchability and Embryonic Mortality

One thousand eight hundred eggs of the Mandara chicken strain were used in 3 batches of hatching at 38, 44, and 50 wk of age (600/batch) (150 eggs/treatment). Daily gathering of eggs was carried out and stored at 16 to 18 °C for a maximum of 5 d before being placed in the incubator. Evaluations of hatchability, embryonic mortality, and fertility were conducted. All hatchlings, dead or alive, were counted when they hatched. The unhatched eggs were analyzed and determined to be either infertile or to have died during embryonic development. Three categories of embryonic mortality were identified by Yakimenko et al. (2002): embryos that have been pipped, late-dead, and early-dead. Fertility is measured as the percentage of viable eggs to whole eggs laid. Hatchability was determined by dividing the number of hatchlings by the number of fertile eggs.

### Hormonal Levels and Reproductive Organs in Males and Females

Nine birds from every group in the study (the same nine hens that were previously slaughtered plus three cocks) were slaughtered by severing their jugular veins at the age of 50 wk following the experiment. Blood was obtained from each bird and placed in non-heparinized pipes. Blood serum was separated by centrifuging the mixture for 15 min at 1107 g. Blood serum was stored at −20 °C for analysis (Dhurat and Sukesh, 2014). Using ELIZA kits from DiaMetra, Spello-Perugia, Italy, serum concentrations of testosterone (T) and estradiol-17β (E2) have been measured. After each bird had been slaughtered, its abdominal cavity opened up. The ovary and oviduct were weighted as soon as they were removed. The number of yellow ovarian follicles was counted, and each female’s oviduct length was measured. Additionally, the right and left testicles of each male were removed and weighed separately (Gongruttananun, 2011).

### Immunological Parameters

After the experiment, the same nine slaughtered birds were chosen to analyze the lymphatic organ weights (spleen and thymus). Furthermore, serum samples were taken from every group (Shahir et al., 2014), and the hemagglutinin inhibition test, which Alexander and Chettle (1977) described, was used to identify serum antibody titers against the IBV and AI to assess the humoral immune response.

### Economic Efficiency

Established on the price of the diets tested and eggs yielded, the input–output examination was utilized to calculate the EE of MOS supplementation in diet and egg production. The values of EE were calculated using the net revenue for each unit of total expenses. This is the methodology used to calculate net revenue and economic effectiveness: Net revenue, measured in Egyptian pounds, is the total cost of all produced eggs minus all feed expenses. The ratio of net revenue to feed intake is used to compute EE. The price of labor, housing, veterinary upkeep, and the purchase price of the birds were not involved, for these expenses were equal for whole treatments.

### Statistical Analyses

The data in SPSS was tested utilizing a General Linear Model (SPSS, 2019). Variances in the mean were evaluated utilizing Duncan’s multiple-range test (Duncan, 1955). A one-way analysis of variance (ANOVA-test) was carried out utilizing the model that follows:


$$\begin{array}{l} Y_{ij}=\mu +T_{i}+e_{ij}; \end{array}$$

where Yij = the observation on the jth separate from the ith treatments. μ = the total mean. Ti = the fixed impact of the ith treatments (Control and MOS treatments). eij = the random error linked with the individual ij.

## Results

### Live Body Weight and Egg Production Traits

The data analysis showed significant differences (*P* ≤ 0.001) between treatment groups for EN, EM, LR, and FCR (Table 2). Interestingly, EW and FC did not differ significantly between the groups. Compared to the control group, using MOS at different levels significantly improved EN, EM, LR, and FCR in Mandarah hens. Notably, hens fed MOS at 0.2 g/kg diet had the best EN, EM, and LR results. Additionally, all MOS groups displayed a better FCR compared to the control.

**Table 2.** Impacts of dietary mannan oligosaccharides supplementation on egg production performance of laying hens (means ± SE)

Traits	Treatments (g/kg diet)				*P*-value
	Control	MOS 0.1	MOS 0.2	MOS 0.5	
Egg number/hen	53.40^b^ ± 0.67	68.66^a^ ± 0.44	69.26^a^ ± 0.82	64.10^a^ ± 0.91	0.008
Egg weight (g)	47.17 ± 0.63	49.08 ± 0.38	48.68 ± 0.50	48.06 ± 0.42	0.086
Egg mass (g /hen)	2,523.23^b^ ± 3.10	3,370.70^a^ ± 3.01	3,374.10^a^ ± 2.32	3,080.03^a^ ± 2.38	0.008
Laying rate	47.67^b^ ± 2.78	61.30^a^ ± 2.40	61.84^a^ ± 2.52	57.23^a^ ± 2.60	0.008
Feed consumption (g/bird)	13,850.25 ± 2.74	13,815.90 ± 2.66	13,842.42 ± 2.31	13,808.33 ± 1.47	0.522
Feed conversion (feed/egg mass)	5.54^a^ ± 0.39	4.09^b^ ± 0.23	4.12^b^ ± 0.21	4.50^b^ ± 0.20	0.010

^a, b^Means within the same row with different superscripts are significantly different (*P* ≤ 0. 001).

**Figure 1.**
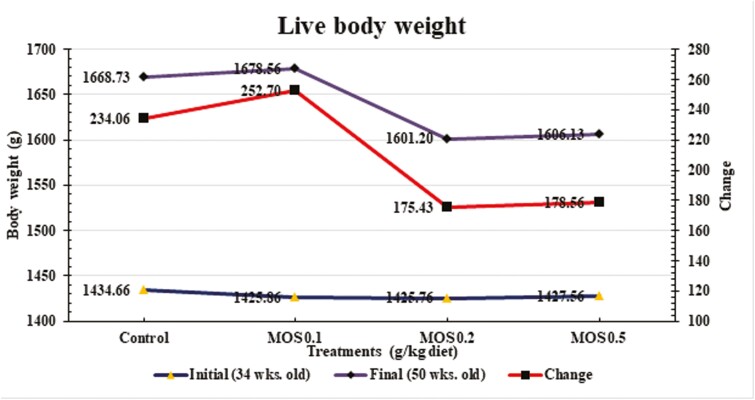
Effects of dietary mannan-oligosaccharides supplementation on live body weight of Mandarah laying hens.

Additionally, Figure 1 did not reveal any statistically significant differences between the treatment groups for final body weight (FBW), change in body weight (CBW), or initial body weight.

### Egg Quality Traits

The impact of dietary MOS supplementation on measures of egg quality in Mandarah laying hens is displayed in Table 3. Treatments did not differ statistically substantially in the ESI, yolk index, or shell thickness, according to analysis of variance, but they did differ significantly in the HU. The highest HU scores were in eggs produced by chickens fed diets containing 0.2 and 0.5 g/kg of MOS. Nonetheless, the control bird’s eggs had the lowest values.

**Table 3.** Impacts of dietary mannan oligosaccharides supplementation on egg quality characteristics of laying hens from 34 to 50 wk of age (means ± SE)

Traits	Treatments (g/kg diet)				*P*-value
	Control	MOS 0.1	MOS 0.2	MOS 0.5	
Egg shape index	75.48 ± 0.76	76.92 ± 0.78	76.71 ± 0.74	76.24 ± 0.73	0.710
Yolk index	44.24 ± 0.82	45.83 ± 0.12	43.91 ± 0.46	43.19 ± 0.91	0.106
Haugh unit score	84.86^b^ ± 0.67	86.99^ab^ ± 0.85	88.98^a^ ± 0.71	89.63^a^ ± 0.61	0.049
Shell thickness (µm)	275.00 ± 0.01	287.34 ± 0.30	280.51 ± 0.04	287.36 ± 0.01	0.350

^a, b, c^Means within the same row with different superscripts are significantly different (*P* ≤ 0. 05).

### Carcass Characteristics

Comparative carcass weight, total edible components weight percent, and abdominal fat (g, %) were found to differ significantly (*P* < 0.05, *P* < 0.001), according to an analysis of variance. The data in Table 4 demonstrated that compared to the control group, the average relative weights of carcass and total edible parts were higher in hens-fed diets containing 0.5 and 0.2 g MOS /kg. Additionally, compared to the control group, chickens fed diets supplemented with MOS at all levels had the lowest mean of absolute and relative abdominal fat weight.

**Table 4.** Impacts of dietary mannan oligosaccharides supplementation on carcass characteristics of laying hens at 50 wk of age (means ± SE)

Traits		Treatments (g/kg diet)				*P*-value
		Control	MOS 0.1	MOS 0.2	MOS 0.5	
Carcass	g	1,036.66 ± 2.62	1,073.33 ± 2.52	1,096.66 ± 2.33	1,108.33 ± 2.04	0.480
	%	62.13^c^ ± 1.12	63.97^bc^ ± 1.42	68.41^ab^ ± 1.92	69.02^a^ ± 1.91	0.021
Giblets	g	88.67 ± 2.36	89.06 ± 2.65	95.49 ± 2.05	87.78 ± 2.88	0.833
	%	5.30 ± 1.46	5.31 ± 1.20	5.99 ± 1.70	5.46 ± 1.10	0.662
Total edible parts	g	1,125.34 ± 2.38	1,162.39 ± 2.12	1,192.16 ± 2.72	1,196.11 ± 2.81	0.419
	%	67.44^b^ ± 1.57	69.28^b^ ± 1.21	74.4^a^ ± 1.31	74.49^a^ ± 1.42	0.010
Abdominal fat	g	175.66^a^ ± 2.53	123.33^b^ ± 2.37	134.33^b^ ± 2.25	127.21^b^ ± 2.64	0.000
	%	10.56^a^ ± 0.68	7.35^b^ ± 0.37	8.39^b^ ± 0.34	7.90^b^ ± 0.06	0.004

^a, b, c^Means within the same row with different superscripts are significantly different (*P* ≤ 0.001).

### Fertility, Hatchability, and Embryonic Mortality

**Figure 2.**
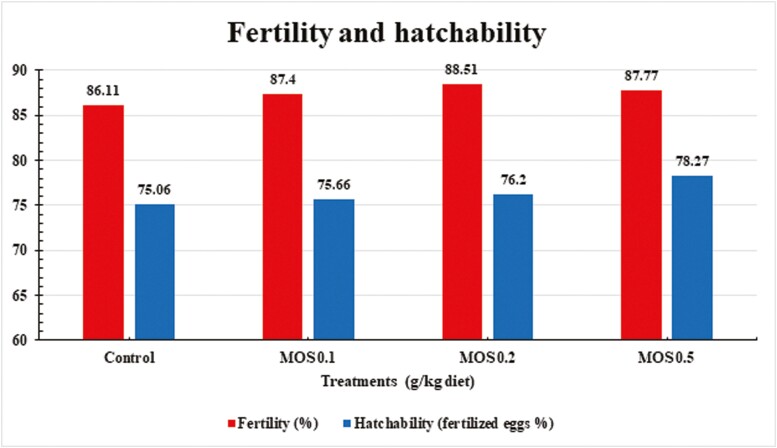
Effects of dietary mannan-oligosaccharides supplementation on fertility and hatchability of Mandarah laying hens.

**Figure 3.**
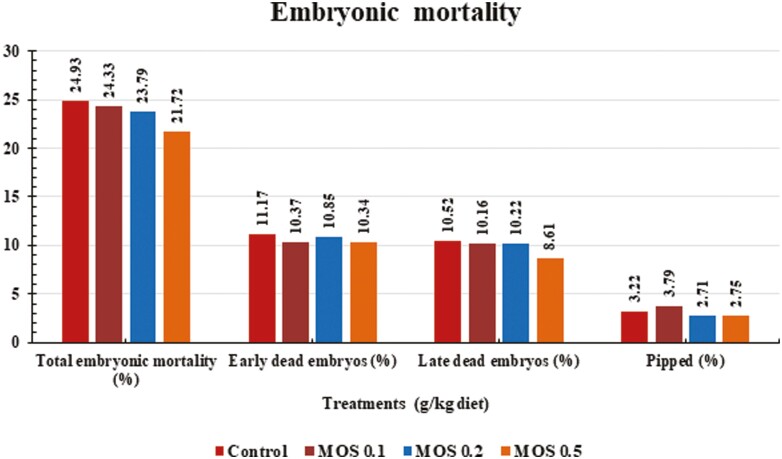
Effects of dietary mannan-oligosaccharides supplementation on embryonic mortality of Mandarah laying hens.

According to the analysis of variance, there were no significant differences between treatment groups in terms of the percentages of early, late and pipped embryos, fertility, and hatchability (Figures 2, 3).

### Hormonal Levels and Reproductive Organs in Males and Females

**Figure 4.**
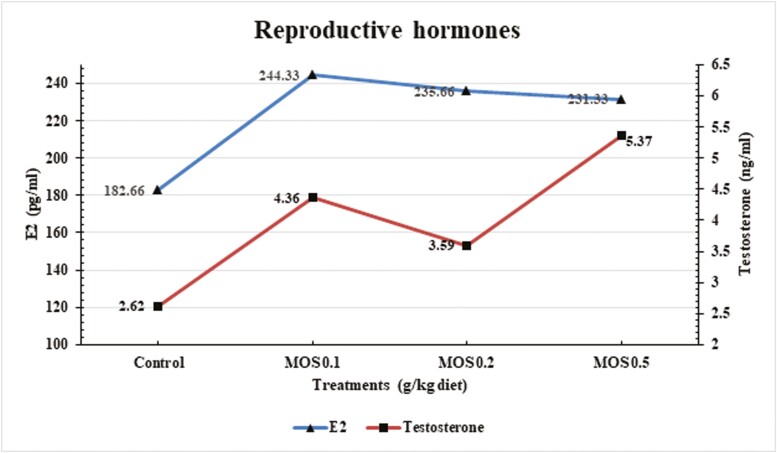
Effects of dietary mannan-oligosaccharides supplementation on estradiol-17β and testosterone levels of Mandarah laying hens at 50 wk of age.

The results of the analysis of variance indicated that the serum concentrations of testosterone and estradiol-17β differed significantly (*P* ≤ 0.001) between the groups that received dietary supplements (Figure 4). The findings showed that compared to the control group, the serum concentricity of estradiol-17β was significantly superior in females fed MOS at all levels. Additionally, the cocks that received MOS at a level of 0.5 g/kg diet were assessed to have the greatest serum concentricity of testosterone. However, the control hens had the smallest concentration.

Statistical analysis revealed no significant changes in several parameters following the treatments (Table 5). These parameters included female body weight (g), ovary weight (g and %), number of yellow follicles in the ovaries, oviduct length (cm), male body weight (g), and total testicular weight (g and %).

**Table 5.** Impacts of dietary mannan oligosaccharides supplementation on females and males reproductive organs of Mandarah laying hens at 50 wk of age (means ± SE)

Traits	Treatments (g/kg diet)				*P*-value
	Control	MOS 0.1	MOS 0.2	MOS 0.5	
*Females*					
Body weight (g)	1,668.66 ± 2.74	1,678.56 ± 2.12	1,601.20 ± 2.86	1,606.13 ± 2.93	0.093
Ovary (g)	35.57 ± 2.05	40.36 ± 2.22	31.24 ± 2.89	47.62 ± 2.61	0.484
Ovary (%)	2.13 ± 0.17	2.40 ± 0.32	1.97 ± 0.37	2.97 ± 0.22	0.438
Ovarian yellow follicles (No.)	3.66 ± 0.16	4.66 ± 0.88	4.33 ± 0.83	4.33 ± 0.16	0.717
Oviduct (g)	57.28 ± 1.92	37.29 ± 2.12	52.00 ± 2.25	39.54 ± 2.23	0.489
Oviduct (%)	3.42 ± 0.46	2.21 ± 0.37	3.26 ± 0.58	2.47 ± 0.43	0.502
Oviduct Length (cm)	62.66 ± 1.91	54.66 ± 1.65	62.00 ± 2.25	56.66 ± 1.56	0.481
*Males*					
Body weight (g)	2,170.66 ± 2.35	2,256 ± 2.27	2,015.33 ± 3.78	2,223.66 ± 2.82	0.698
Total testes weight (g)	19.89 ± 1.39	29.12 ± 1.91	29.85 ± 1.43	24.49 ± 1.87	0.268
Total testes weight (%)	0.92 ± 0.08	1.29 ± 0.21	1.47 ± 0.04	1.09 ± 0.08	0.065

### Immunological Parameters

**Figure 5.**
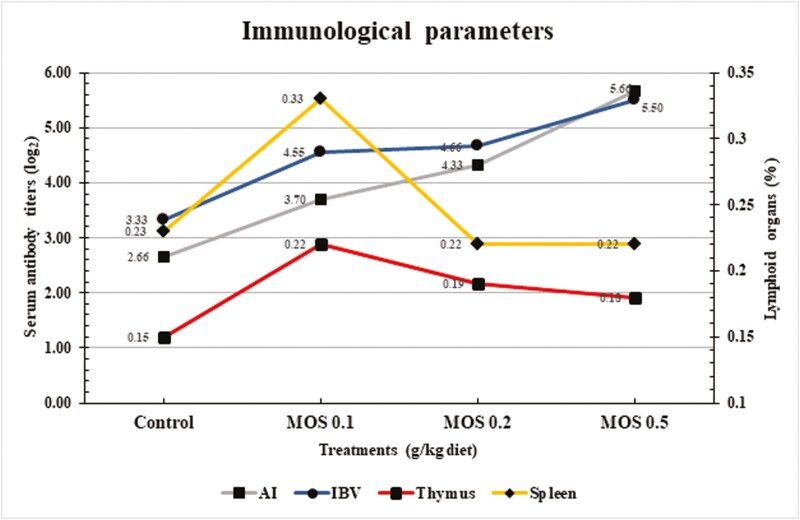
Effects of dietary mannan-oligosaccharides supplementation on Mandarah laying hens’ serum antibody titers and lymphoid organs at 50 wk of age.

As shown in Figure 5, adding MOS to the feed impacted the immune response of the birds at the end of the experiment. The figure reveals significant differences (*P* < 0.05, 0.001) between the MOS and control groups in several immune parameters. These parameters include serum antibody levels against the H9N2 influenza virus and IBV and the weight percentage of lymphoid organs (spleen and thymus). Also, chickens fed diets containing 0.5 and 0.2 g of MOS per kg had significantly higher levels of antibodies in their blood against IBV and H9N2 compared to the control group. Additionally, the groups had significant differences (*P* < 0.05) in the relative weights of the thymus and spleen. Interestingly, hens fed diets with only 0.1 g of MOS/kg also showed a significant increase in spleen and thymus weight compared to the control group.

### Economic Efficiency

As indicated in Table 6, the net revenue over the diet price measures economic effectiveness. When Mannan was compared to the control group in the present experiment, economic effectiveness was raised and the relation between economic effectiveness advanced at all concentrations. Also, regarding relative EE, utilizing MOS at a 0.1 g/kg diet outperformed all other MOS. As illustrated in Table 6, the relative EE for MOS at levels of 0.1, 0.2, and 0.5 g/kg diet was 343.69%, 334.68%, and 208.35%, respectively, compared to the control group (100%).

**Table 6.** Input–output analysis and economic efficiency of the experimental groups

Traits	Treatments (g/kg diet)			
	Control	MOS 0.1	MOS 0.2	MOS 0.5
Number of eggs/hen	53.4	68.66	69.26	64.1
Eggs price (LE)^*^	93.45	120.16	121.21	112.18
Feed consumption (kg)	13.85	13.82	13.84	13.81
Feed cost (LE)^†^	83.1	84.14	85.55	89.06
Net revenue/hen (LE)^‡^	10.35	36.016	35.659	23.111
Economic efficiency (EE)^‖^	12.45	42.8	41.68	25.94
Relative economic efficiency (%)^$^	100	343.69	334.68	208.35

^*^Total egg number × 1.75 (one egg 1.75 L.E).

^†^Total feed consumption × price (the price of 1 kg of control feed was 6.00 pounds). The price of one kilogram of mannan was 900 pounds. The price of a kilogram of diet plus 0.1, 0.2, and 0.5 g of mannan was 6.090, 6.180 and 6.450 L.E, respectively.

^‡^Net revenue/hen (LE) = price of all eggs/hen (LE) minus total feed cost/hen.

^‖^Economical efficiency = net revenue/feed intake price × 100.

^$^Economic efficiency for treatment/economic efficiency of control × 100.

## Discussion

The present study found that using MOS at different doses increased egg production in laying hens. This was shown by an improvement in several factors, including the number of eggs laid, the weight of the eggs, and the rate at which they were laid. Hens-fed MOS also had a better FCR than the control group.

These results are in agreement with those described by Güçlü (2011), Bozkurt et al. (2012), Jahanian and Ashnagar (2015), Hassan et al. (2015) and Salem and El-Dayem (2024), who found that feeding mannan to laying hens increased their EP, EM, and FCR. Additionally, these findings support those made by Berry and Lui (2000), Stanley et al. (2000), Gürbüz et al. (2011) and Bozkurt et al. (2012), who discovered that mannan supplementation held no impact on the weight of the egg. In this context, MOS act as modulators of the intestinal microbiome, which is essential for the body’s immune system to activate, digestive enzymes to activate, nutrients to be better absorbed from feed by eliminating pathogenic bacteria, and toxins secreted by pathogens to be neutralized (Szymaska-Czerwiska and Bednarek, 2008). The involvement of MOS in boosting gastrointestinal enzyme activity, gastrointestinal microbiota, and intestinal shape may be responsible for these enhancements in digestion and nutrient absorption (Yang et al., 2008). Additionally, Berry and Lui (2000) observed a significant increase in EP inside the MOS-fed chickens. This might be because MOS eliminates bacteria from the stomach after type-1 fimbriae from distinct bacterial strains adsorb harmful bacteria (Spring et al., 2000).

Additionally, MOS’ capacity to inhibit the development of potentially dangerous bacteria in animals’ digestive tracts contributes to their ability to promote growth (Bozkurt et al., 2008; Limbu et al., 2024). As a result, the digestive system stays in good condition, performs better, and more nutrients are available for absorption. Also, Rizwan et al. (2016) report that the MOS is useful for chickens, as it improves slaughter performance and reduces vulnerability to gastrointestinal and respiratory diseases. Because they are not wrecked down in the stomach, MOS in the small intestine stays uninterrupted and promotes intestinal microbiota action (Shao et al., 2013). Prebiotics improve the body’s ability to absorb macro- and micronutrients, making them more available (Chen and Chen, 2004; Korkach et al., 2024).

Significant variations were in the overall mortality rate, but no statistically significant distinctions between the experimental groups for FBW and CBW were found. These results corroborate those of Stanley et al. (2000), Gürbüz et al. (2011), and Bozkurt et al. (2012), who discovered no influence on FBW was observed when using MOS in laying hen diets. In contrast, the FBW of layer hens given MOS was considerably superior to that of the control group raised in exceptionally hot climate conditions (Cabuk et al., 2006). MOS decreased the mortality rate of chickens. This enhancement in viability may be linked to a rise in immunity and resilience of treated birds against illnesses.

Additionally, MOS increased the production of useful microorganisms and decreased the amount of harmful microorganisms, resulting in a healthy intestinal situation (Joseph et al., 2023). This improved the intestines’ ability to absorb nutrients, which improved function. Additionally, a decrease in immunity and resistance to disease may linked to the rise in mortality in the control group (OKEY, 2023).

The HU score remains the most common method professionals employ to assess the quality of the albumen and, consequently, the freshness of an egg (Sehirli and Arslan, 2022). When MOS-supplemented diets were given to laying hens, the results showed higher HU scores at levels 0.2 and 0.5 g/kg diet. Moreover, albumin quality was quantitatively improved in MOS groups, which may suggest MOS contributes to egg freshness. Also, MOS supplementation in laying hens increased the HU score, which is consistent with these findings (Jahanian and Ashnagar, 2015). Our results are similar to those of other studies. Bozkurt et al. (2012) and Abd El-Samee et al. (2012) found no significant effect of adding MOS to chicken feed on the shape or yolk index of the eggs. Likewise, hens fed a diet with added yeast showed no differences in the quality of either the egg albumen or yolk (Nursoy et al., 2004). MOS may improve gut health by influencing digestive enzymes, gut bacteria, and the shape of the intestines. These changes might lead to better nutrient absorption and use by the hens (Gurbuz et al., 2011; Aziz et al., 2024).

Furthermore, the study demonstrated that MOS treatments improved the carcass characteristics of laying hens. These results are consistent with those reported by Abd El Latif (2023), who discovered that feeding broiler chickens mannan and/or commercial enzymes improved the carcass parameters. Additionally, Kwiecień et al. (2023) discovered that adding l-carnitine and Mos to turkey feed improved the flexibility and fracture toughness of the femur while positively influencing several rearing indices and carcass quality.

MOS can improve growth and feed conversion efficiency in poultry by lowering illness and fostering intestinal health. This may result in a larger carcass that yields more meat. Additionally, some research indicates that MOS may enhance the quality of meat by lowering oxidative stress and enhancing muscle integrity, which could result in more delicate and tasty meat. Additionally, supplementing with MOS has been associated with marginally reduced fat deposition in chickens, which may lead to slimmer carcasses (Simmons et al., 2023). Moreover, MOS function as prebiotics, favorably stimulating the development of advantageous gut flora such as *Bifidobacteria* and *Lactobacilli*. These good bacteria reduce pathogenic bacteria’s colonization and potential harm to gut health by competing with them for nutrients and attachment sites. The increased gut health may indirectly impact the accumulation and metabolism of fat. In the intestines, certain MOS structures can bind to bile acids and cholesterol, possibly increasing their excretion and decreasing their absorption. Ultimately, this might result in less fat deposited (Tian et al., 2023).

Moreover, data also showed no significant effect of MOS treatments on fertility, hatchability and embryo death rates (early, late, and pipped). Hatchability is known to be influenced by several factors. How well chicks hatch depends a lot on the health and diet of the breeding birds, as well as the size, weight, and overall quality of the eggs they lay. Weight, shell thickness, and shape are the most important factors in egg quality for hatching (King’ori, 2011). Shashidhara and Devegowda (2003) state that MOS boosts broiler breeders’ fertility and hatchability. Also, diets with Bioplex Zinc reaching 40 mg/Kg only or in combination with 1.0 g MOS/Kg considerably increased the hatchability of quail eggs.

Furthermore, the results showed that utilizing MOS, especially at 0.1 g/kg diet, enhanced female reproductive qualities when demonstrated through a rise in serum E2 concentration. Estradiol-17β was a key player in female sexual and reproductive behavior, although its levels fluctuated according to a person’s physiological state (Pfaus et al., 2003). Additionally, elevated E2 levels and female reproductive characteristics were found to be highly correlated by Ilès et al. (2013). Instead, testosterone concentrations were greater after MOS supplementation at a 0.5 g/kg diet than in the control group. This result makes sense when one considers that when the gut microbial community becomes unbalanced because of stress or diet, bacteria such as those that cause endotoxemia and those that are Gram-negative pose a major risk to the host’s health (Tremellen, 2016; Zaher et al., 2024). Thus, the possibility that prebiotics and probiotics could reduce endotoxins in the blood and alleviate their aftereffects, including inflammation and other undesirable health markers, could be the reason behind the rise in plasma testosterone concentration (Gurung, 2017).

The current study shows that giving MOS in different amounts to Mandarah hens boosted their immune system. Hens given MOS had higher antibodies in their blood against H9N2 and IBV viruses. Additionally, their lymphoid organs, which are important for immunity, were in better condition compared to the hens that did not get MOS. These findings are consistent with previous studies (Sheikh et al., 2020; Youssef et al., 2023c). MOS act as prebiotics, selectively feeding beneficial gut bacteria like *Lactobacilli* and *Bifidobacteria*. These bacteria produce short-chain fatty acids (SCFAs) that stimulate gut health and immune function. SCFAs also modulate regulatory T cells, promoting immune tolerance and reducing inflammation, potentially leading to increased resource allocation for lymphoid organ growth (Perini et al., 2023). Also, MOS exhibits anti-inflammatory properties by reducing pro-inflammatory cytokine production and enhancing antioxidant activity. This reduced inflammation allows for better resource allocation towards immune system development and the growth of lymphoid organs (Yang et al., 2023). Also, some studies suggest MOS might directly interact with the lymphatic system, enhancing the absorption and transport of immune cells and immune-modulating molecules, contributing to improved immune function and potentially larger lymphoid organs and increased antibody production (Wlazlak et al., 2023; Baek et al., 2024).

The findings of Hassan and Ragab (2007) and Bahakaim et al. (2015), who found that adding MOS to laying hens’ diets produced the highest relative economic efficiencies, align with these results. At all MOS levels, the current study discovered that relative EE was higher than in the control set. This improvement could result from increased egg quantity, EM, LR, and FCR (Salem and El-Dayem, 2024).

## Conclusion

This study demonstrated that Mandarah chickens fed mannan-oligosaccharides (MOS) showed significant improvements in their laying performance, carcass quality, immune response and overall profitability compared to control birds. These results suggest that MOS could be a valuable feed additive, potentially replacing antibiotics to boost egg production in laying hens.
